# Supplementary material for: Comparative genomics provides new insights into the diversity, physiology, and sexuality of the only industrially exploited tremellomycete: Phaffia rhodozyma
Source: BMC Genomics. 2016 Nov 9;17:901. doi: 10.1186/s12864-016-3244-7 (PMC5103461; doi:10.1186/s12864-016-3244-7)
Supplement: Additional file 6: — List of orphan genes with links to PFAM (related to Additional file 1: Table S1). (ZIP 1428 kb) [file 12864_2016_3244_MOESM6_ESM.zip › PFAM_UNIQUE/G01859_P.html]

#                                                                            --- full sequence --- -------------- this domain -------------   hmm coord   ali coord   env coord
  
# target name        accession   tlen query name           accession   qlen   E-value  score  bias   #  of  c-Evalue  i-Evalue  score  bias  from    to  from    to  from    to  acc description of target
  
#------------------- ---------- ----- -------------------- ---------- ----- --------- ------ ----- --- --- --------- --------- ------ ----- ----- ----- ----- ----- ----- ----- ---- ---------------------
  
G01859\_P             -            276 FSIP1                PF15554.1    425       8.6    3.7   8.1   1   1     0.023        14    3.0   8.1    19   105   147   235   136   266 0.54 - FSIP1 family  
G01859\_P             -            276 Tir\_receptor\_C       PF07489.6    221       2.4    6.5  11.7   1   1    0.0015       1.5    7.2   9.6    84   150   137   203    96   228 0.66 - Translocated intimin receptor (Tir) C-terminus
